# Primary Hypothyroidism with Pituitary Hyperplasia in an Omani Girl

**DOI:** 10.1155/2022/3382612

**Published:** 2022-05-29

**Authors:** Wafa Abdallah Fadle, Ali Al Reesi, Saud Al-Shabibi, Maryam Khamis Al-Badi

**Affiliations:** ^1^Department of Pediatric Endocrinology, National Diabetes and Endocrine Center, Royal Hospital, Muscat, Oman; ^2^Department of Internal Medicine, Sohar Hospital, Sohar, Oman; ^3^Department of Radiology, Royal Hospital, Muscat, Oman

## Abstract

Pituitary hyperplasia secondary to primary hypothyroidism (PHPH) is uncommon in children and is reversible with thyroxine therapy. We report an Omani girl who presented at the age of 13 years and 6 months with profound primary hypothyroidism due to Hashimoto's thyroiditis and secondary pituitary hyperplasia and hyperprolactinemia. Pituitary magnetic resonance imaging confirmed the presence of pituitary hyperplasia which regressed during follow-up after the administration of thyroxine therapy. The diagnosis of PHPH is very important in both children and adults in order to avoid unnecessary brain surgery or medical treatment for a presumed pituitary mass or adenoma. To our knowledge, this patient represents the first case of an Omani child presenting with PHPH.

## 1. Background

Primary hypothyroidism is a common endocrine disorder in children [1]. Hashimoto's thyroiditis is the most common cause of hypothyroidism in children; according to recent epidemiological data, the prevalence of primary hypothyroidism (autoimmune thyroiditis) is 0% among children aged 1–6 years, 1.2% among children aged 6–12 years, and 3.2% among children aged 12–16 years [2]. Long-standing primary hypothyroidism in children with high serum levels of thyroid-stimulating hormone (TSH), and the subsequent proliferation of TSH-releasing cells, may lead to pituitary hyperplasia, causing what is known as pituitary hyperplasia secondary to primary hypothyroidism (PHPH) [1]. PHPH was first reported in adults by Niepce in 1851, with similar cases reported thereafter [3–5]. However, PHPH is rarely reported in children; a recent review identified only 18 cases of pediatric PHPH in the literature [1]. Here, we present an interesting case of an Omani female adolescent who presented with a history of severe headache and short stature at the age of 13 years and 6 months and who was subsequently found to have an enlarged pituitary gland secondary to profound primary hypothyroidism.

## 2. Case Report

### 2.1. Patient History

An Omani girl aged 13 years and 6 months presented to a local hospital with a history of headache and was seen by an endocrinologist. She was found to have abnormal thyroid function test findings indicative of primary hypothyroidism and high prolactin levels; accordingly, she was started on thyroxine therapy and referred to the National Diabetes and Endocrine Center, Muscat, Oman. Her initial symptoms consisted of a two-year history of edema of the face, hands, and feet associated with cold intolerance and poor academic performance. In addition, she complained of recurrent headaches over the preceding 6 months. There were no symptoms of vomiting, blurred vision, seizure, changes in bowel habits, dysphagia, dysphonia, hair loss, or chest symptoms; however, she suffered from fatigue throughout the day. There was no family history of similar issues, short stature, or autoimmune disorders. Her developmental history was normal. There was no history of surgery and she was not currently taking any long-term medications.

### 2.2. Physical Examination

**Figure 1:**
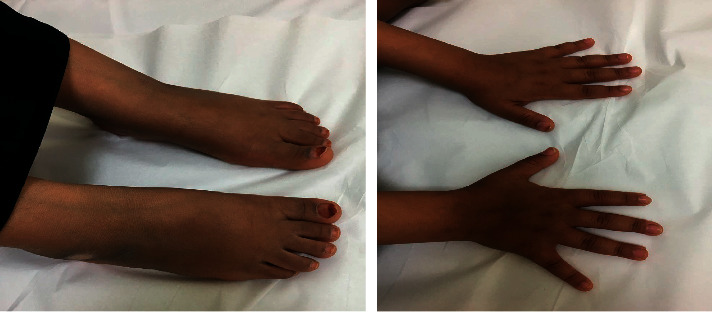
Photographs showing edema of both hands and feet.

The patient was 127 cm in height, had a body weight of 33.9 kg, and had a midparental height of 145 cm. Based on the criteria of the World Health Organization, she was below the 3^rd^ percentile for both height (−4.8 cm SD below the mean) and midparental height (4.1 cm SD below the mean) and within the 3^rd^ percentile for body weight. Her body mass index was 21.01 kg/m^2^, conforming to the 95^th^ centile. Her pulse rate was 77 beats per minutes, and her blood pressure was 100/63 mmHg. During the physical examination, the patient was found to have a small, diffuse, palpable thyroid gland with no palpable nodules or lymph nodes. There was visual evidence of facial puffiness and edema in both hands and feet (Figure 1), without calf muscle hypertrophy. In terms of sexual development, the patient had breasts and pubic hair (Tanner stage 3) but had no axillary hair and had not yet attained menarche. There was normal speech and limb activity. A neurological examination was normal. She was also seen by an ophthalmologist who confirmed that there was no papilledema.

### 2.3. Laboratory Findings

Basic biochemical findings indicated low free thyroxine (FT4) levels (<0.8 pmol/L; normal range: 8.5–22.5 pmol/L), high TSH levels (>100 mIU/mL; normal range: 0.35–5 mIU/L), high thyroglobulin antibody levels (76.1 IU/mL; normal range: <0.4 IU/mL), and high anti-thyroid peroxidase antibody levels (560.8 IU/mL; normal range: <6 IU/mL). In addition, her prolactin level was high initially (3,173 IU/mL; normal range: 102–496 IU/mL). Levels of insulin-like growth factor (IGF)-1 (21.8 nmol/L; normal range: 18.6–68.8 nmol/L) and IGF-binding protein 3 (3.9 mg/L; normal range: 3.1–9.5 mg/L) were in the low end of normal range. All other laboratory investigations including liver function test, renal function test, bone profile, and cortisol levels were normal, apart from a lipid profile which showed high levels of low-density lipoprotein cholesterol (4.49 mmol; normal range: <2.7 mmol) and total cholesterol (5.82 mmol; normal range: 3.2–5.18 mmol), with normal levels of high-density lipoprotein cholesterol. The patient was also investigated for puberty markers, including luteinizing hormone, follicle-stimulating hormone, and estradiol levels, all of which were found to be in the normal pubertal range.

### 2.4. Radiological Examination

**Figure 2:**
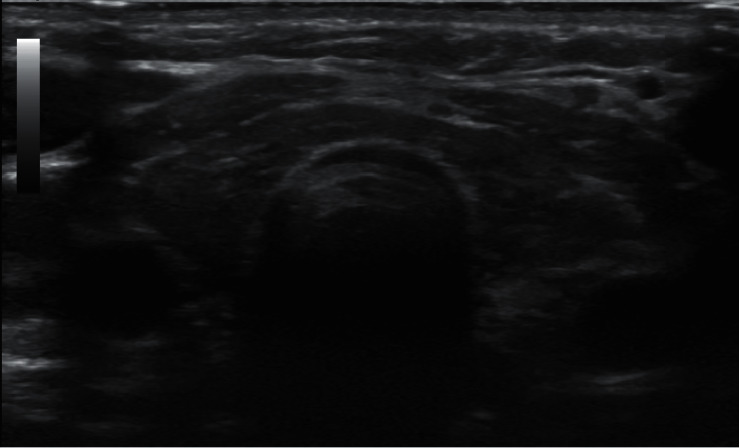
Thyroid ultrasound showing the relatively small size of the lobes and isthmus, diffuse coarse, heterogenous parenchymal echotexture with multiple fibrous echogenic lines, and no focal nodule.

**Figure 3:**
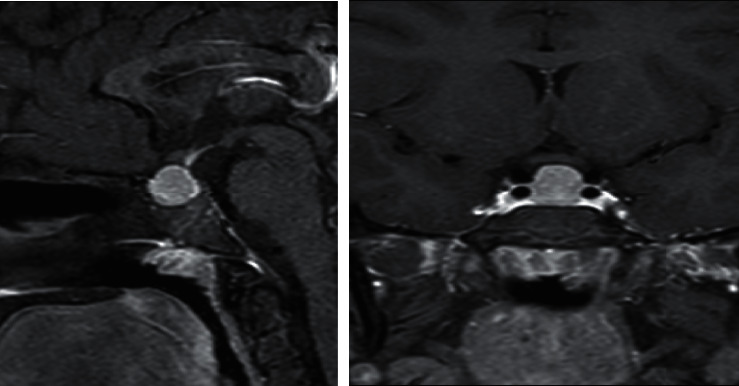
Sagittal and coronal contrast-enhanced T1-weighted magnetic resonance images taken at presentation. The images showed a homogenously enhancing solid mass arising from the pituitary gland with a superior concave border abutting the optic chiasm. There was no cystic/necrotic, haemorrhagic, or calcific component.

**Figure 4:**
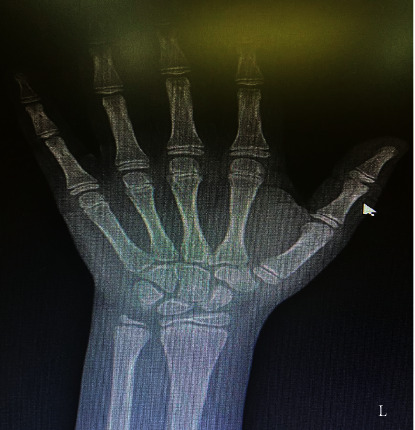
Left wrist X-ray showing a bone age of 10 years in contrast to chronological age of 13.6 years. Bone age was determined using Greulich and Pyle's radiographic atlas of skeletal development of the hand and wrist [6].

An ultrasound of the thyroid showed that both lobes and isthmus were relatively small in size, with diffuse, coarse, heterogenous parenchymal echotexture with multiple fibrous echogenic lines and no focal nodule (Figure 2). As the patient complained of severe headaches, pituitary magnetic resonance imaging (MRI) was performed, revealing a large, nearly homogenous lesion replacing the pituitary glands, with an isointense signal on T1- and T2-weighted images with widening of the *sella turcica* and superior displacement of the pituitary stalk (Figure 3). The lesion measured approximately 1.3  × 1.4 × 1.4 cm (anteroposterior x transverse x coronal). The T1-weighted images showed high signal intensity of the posterior pituitary glands. There was no significant evidence of necrosis or hemorrhage within the lesion. The radiological examination of the rest of the brain was unremarkable. Despite the child's chronological age being 13 years and 6 months, her bone age was estimated to be 10 years (Figure 4), as per Greulich and Pyle's radiographic atlas of skeletal development of the hand and wrist [6]. A repeated left wrist X-ray at the age of 16 years and 2 months showed a delayed bone age of 13 years.

### 2.5. Treatment

The patient was started on 25 *μ*g of thyroxine once daily which was increased gradually according to her thyroid function test results (Table 1). Following the administration of thyroxine, her prolactin levels decreased until they normalized. Due to the patient's short stature, a growth hormone stimulation test was performed once her thyroid function results normalized. Both glucagon and clonidine showed poor response (Tables 2 and 3). The patient was started on growth hormone (GH) therapy at 30 *μ*g/kg/day and showed good response, with a growth velocity of 8 cm/year in the first year. She received GH therapy for two years, before stopping for one month. During this time, a repeated GH stimulation test using glucagon on her last follow-up visit at the age of 16 years and 2 months showed poor response, with a peak of 6.33 mIU/L (normal range: >20 mIU/L). The GH therapy was therefore reinitiated.

### 2.6. Follow-Up

**Figure 5:**
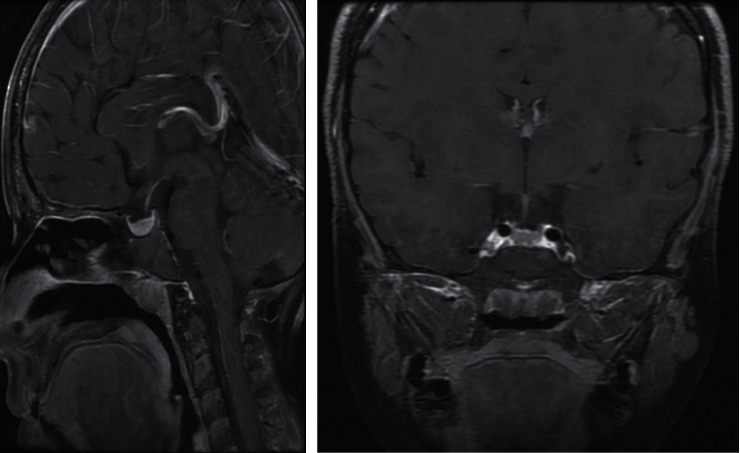
Sagittal and coronal contrast-enhanced T1-weighted magnetic resonance images taken at follow-up after 6 months of thyroid hormone replacement therapy. The images showed a moderate interval reduction in the size of the pituitary mass.

**Figure 6:**
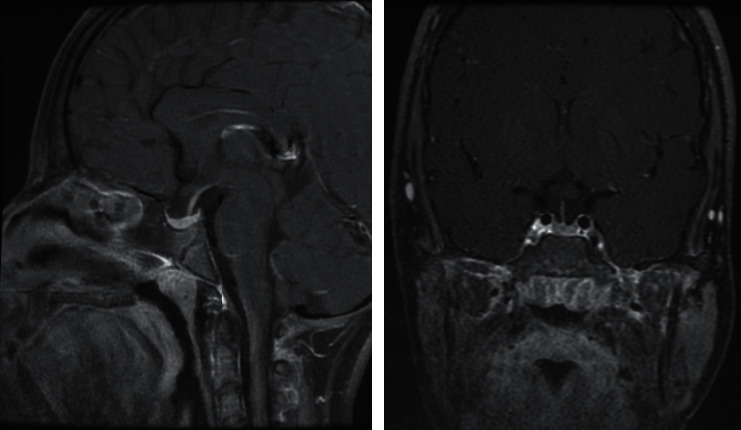
Sagittal and coronal contrast-enhanced T1-weighted magnetic resonance images taken after two years of follow-up. The images showed significant improvement of the pituitary mass as demonstrated by the marked reduction in size, returning to a normal appearance.

The patient was followed up every three months and continued taking thyroxine in combination with GH therapy, demonstrating good clinical and biochemical response with regards to thyroid function, lipid profile, and prolactin levels (Table 1). Follow-up contrast-enhanced pituitary MRI was performed 6 months after the initiation of thyroxine therapy. The comparison sagittal and coronal T1-weighted images demonstrated a moderate interval reduction in the size of the pituitary mass (now measuring 1.2 × 0.7 × 1.1 cm) (Figure 5). Moreover, at 18 months, follow-up sagittal and coronal contrast-enhanced T1 weighted images showed significant improvement of the pituitary mass with a marked reduction in size and returning to normal appearance (Figure 6). At her last consultation, the patient was aged 16 years and 2 months, her height was 144.8 cm, her midparental height was 145.4 cm, and she was found to be at stage 3 of puberty. She was clinically well and demonstrated normal biochemical features.

## 3. Discussion

Prolonged primary hypothyroidism can lead to pituitary hyperplasia due to the loss of negative feedback caused by a lack of FT4 and triiodothyronine (T3), which leads to excessive secretion of thyrotropin-releasing hormone (TRH) from the hypothalamus [4]. In addition, long-standing stimulation of TRH can promote the proliferation of prolactin-releasing cells, leading to the oversecretion of prolactin [7]. In this case report, we describe a female adolescent with PHPH who presented with a history of headaches and hand and facial edema of almost one year in duration. To our knowledge, this is the first report of an Omani pediatric patient with PHPH in the literature.

The binding of the thyroid hormone to the nuclear receptor stimulates the synthesis of GH. As such, children with PHPH typically exhibit low or no secretion of GH from the pituitary gland after stimulation [8, 9]. A recent literature review identified 18 cases of PHPH in children, of which 13 were found to have elevated prolactin levels and four with decreased GH levels [1]. In the present case, our patient had a high prolactin level which normalized after introducing thyroxine therapy. She also showed low peak GH levels following stimulation tests performed with clonidine and glucagon even, once her thyroid function test results had normalized.

The prevalence of isolated growth hormone deficiency (GHD) in Hashimoto's thyroiditis in adults was found to be 5% according to two studies [10, 11]. Another Dutch study in adults showed the prevalence of GHD in Hashimoto's thyroiditis to be much lower (0.4%) [12]. Our patient was investigated for GHD as she was significantly short in stature, with a height SD of −4.8 cm below the mean. She was started on a small dose of 30 *μ*g/kg/day of GH therapy, resulting in some response in her growth velocity, although this could also have been related to the introduction of thyroxine treatment. However, a repeated GH stimulation test showed poor response; hence, the GH therapy was continued.

Laboratory findings in our patient were similar to those reported in the literature, with high TSH levels and low T4 and T3 levels. She was started on low-dose thyroxine treatment with gradual incrementation; as a result, her symptoms improved dramatically. Her pituitary MRI showed an enlarged pituitary gland with marked regression in size within six months of treatment with biochemical improvement. Based on the literature, regression of a pituitary mass to normal size usually occurs over 2–4 months, ranging from 1 to 18 months [13, 14].

In conclusion, it can be challenging to differentiate PHPH from pituitary adenomas based on the initial clinical presentation and MRI findings. Thyrotropin (TSH)-secreting pituitary adenomas (TSH-omas) are a rare cause of hyperthyroidism. In this situation, TSH secretion is autonomous and refractory to the negative feedback of thyroid hormones (inappropriate TSH secretion), and TSH itself is responsible for the hyperstimulation of the thyroid gland and the consequent hypersecretion of T4 and T3 [15,16] .However, a definitive diagnosis is crucial as treatment varies widely. Pituitary adenomas usually require surgical resection or medical treatment, whereas thyroid hormone replacement therapy is the principal means of treatment of PHPH. As such, an understanding of the differences between these conditions is essential to avoid irreversible complications arising from unnecessary surgical or medical intervention. Repeated brain MRI 6 months after thyroxine therapy can be useful to confirm the diagnosis of PHPH. There is an urgent need for further research to assess the true incidence and prevalence of PHPH in children with Hashimoto's thyroiditis as it is likely that this condition is underdiagnosed.

## Figures and Tables

**Table 1:** Biochemical and radiological findings and thyroid hormone doses at diagnosis and during follow-up.

Variable	Time interval					
	At diagnosis	3 m	6 m	9 m	18 m	Last visit
Age	13 y 6 m	13 y 9 m	14 y	14 y 3 m	15 y	16 y 2 m
TSH (mIU/L)^*∗*^	>100	4.96	3.08	2.1	0.755	0.854
FT4 (pmol/L)^*∗∗*^	0.8	12.4	10.8	10.8	15.4	12.7
LDL (mmol/L)^†^	5.7	3.3	3.5	2.8	2.8	2.8
Prolactin (mIU/mL)^‡^	3,173	444	—	—	261	—
Thyroxine treatment	25 *μ*g	50 *μ*g	75 *μ*g for 4 days/50 *μ*g for 3 days	-	75 *μ*g for 4 days/50 *μ*g for 3 days	75 *μ*g for 4 days/50 *μ*g for 3 days
Wrist X-ray	Bone age of 10 years as per Greulich and Pyle [6]	—	—	—	—	Bone age of 13 years as per Greulich and Pyle [6]
Pituitary MRI	Mass in the sellar and suprasellar region measuring 1.3 × 1.4 × 1.4 cm	—	Mass in the sellar and suprasellar region measuring 1.2 × 0.7 × 1.1 cm	—	—	Significant improvement of the mass in terms of size and appearance

y = year; m = month; TSH = thyroid-stimulating hormone; FT4 = free thyroxine; LDL = low-density lipoprotein; MRI = magnetic resonance imaging. ^*∗*^Normal range: 0.35–5 mIU/L. ^*∗∗*^Normal range: 8.5–22.5 pmol/L. ^†^Normal range: <2.7 mmol. ^‡^Normal range: 102–496 IU/mL.

**Table 2:** Growth hormone stimulation test findings following clonidine intake.

	Time after clonidine intake (minutes)				
	0	30	60	90	120
GH (mIU/L)^*∗*^	0.94	0.89	6.53	3.00	1.3

GH = growth hormone. ^*∗*^Normal range: >20 mIU/L.

**Table 3:** Growth hormone stimulation test findings following glucagon intake.

	Time after glucagon intake (minutes)						
	0	30	60	90	120	150	180
GH (mIU/L)^*∗*^	0.9	0.57	1.19	4.09	1.28	4.31	6.96

GH = growth hormone. ^*∗*^Normal range: >20 mIU/L.
